# Use of Bacterial Artificial Chromosomes in Baculovirus Research and Recombinant Protein Expression: Current Trends and Future Perspectives

**DOI:** 10.5402/2012/628797

**Published:** 2012-09-12

**Authors:** Polly Roy, Rob Noad

**Affiliations:** ^1^Department of Pathogen Molecular Biology, Faculty of Infectious Diseases, London School of Hygiene and Tropical Medicine, London WC1E 7HT, UK; ^2^Centre for Emerging Endemic and Exotic Diseases, Department of Pathology and Infectious Diseases, Royal Veterinary College, Hatfield AL9 7TA, UK

## Abstract

The baculovirus expression system is one of the most successful and widely used eukaryotic protein expression methods. This short review will summarise the role of bacterial artificial chromosomes (BACS) as an enabling technology for the modification of the virus genome. For many years baculovirus genomes have been maintained in *E. coli* as bacterial artificial chromosomes, and foreign genes have been inserted using a transposition-based system. However, with recent advances in molecular biology techniques, particularly targeting reverse engineering of the baculovirus genome by recombineering, new frontiers in protein expression are being addressed. In particular, BACs have facilitated the propagation of disabled virus genomes that allow high throughput protein expression. Furthermore, improvement in the selection of recombinant viral genomes inserted into BACS has enabled the expression of multiprotein complexes by iterative recombineering of the baculovirus genome.

## 1. Baculovirus Protein Expression

### 1.1. Baculoviruses

**Figure 1:**
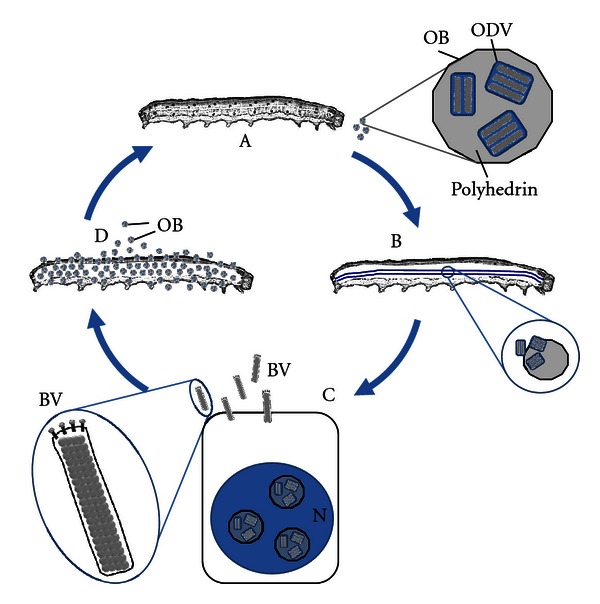
Key stages in the infection cycle of AcMNPV. (A) Infection is initiated by the ingestion of a virus occlusion body (OB). This consists of multiple virus nucleocapsids surrounded by a single lipid envelope (ODV) embedded in a protein matrix formed by the virally encoded polyhedrin protein. (B) The occlusion body is dissolved by the alkaline environment of the insect mid-gut, releasing ODV which initiate a primary infection in the midgut epithelial cells. (C) Virus enters cells and replicates in the nucleus. Two different forms of infectious virus are produced in infected cells. Budded virus (BV) is released at the cell surface and mediates systemic infection of the insect via the tracheal system, and ODV remains embedded in occlusion bodies. (D) Late stages of virus infection trigger liquefaction of the host, releasing the environmentally stable proteinaceous occlusion bodies. Polyhedrin protein is nonessential for the infection of cells in continuous culture in the laboratory and its high level of synthesis makes its promoter ideal for the high-level production of recombinant protein.

The Baculoviridae is a family of viruses with a circular dsDNA genome, ranging in size from 80 kb to 180 kb, that infect arthropods. Virus particles are rod-shaped, with a lipid envelope derived from the host cell. The family is divided into four genera, based on the comparison of a subset of core genes conserved between all baculoviruses [1, 2]. The viruses used for recombinant protein expression are *Autographa californica *multiple nucleopolyhedrosis virus(AcMNPV) and *Bombyx mori *nucleopolyhedrosis virus(BmNPV) and both are in the *Alphabaculovirus* genus. Both viruses are pathogens of the larval stages of lepidopteran species and have an infection cycle that involves ingestion and infection of the cells of the mid-gut, followed by systemic infection. In contrast to vertebrate herpesviruses, which have a similarly sized, circular DNA genome, and which are largely cryptic infections except in immunocompromised individuals, infection of caterpillars with AcMNPV and BmNPV results in a short duration, acute, systemic infection with liquefaction of the host (Figure 1). Once the host is liquefied, the virus remains in the environment encased in a protein matrix formed from the virally encoded polyhedrin protein until it is ingested by the next caterpillar and the alkali environment of the insect mid-gut triggers dissociation of the polyhedrin coat and release of the virus [3, 4]. 

Reflecting the complex nature of their genome, baculovirus infections have a complex multiphase replication cycle that involves a distinct series of transcriptional steps. AcMNPV enters the host cell by a fusion process, mediated by the GP64 protein on the virus surface [5–10], and the nucleocapsid is trafficked to the nucleus. The virus has a genome of 133 894 bp double-stranded (ds) DNA and encodes 154 putative ORFs [11]; following nuclear entry, some of the corresponding genes are transcribed by the host RNA polymerase II and are termed immediate early genes. These genes encode proteins with a range of functions including some that act as transactivators for the expression of other viral genes that start to be produced late (6–12 hours) and very late (16–76 hours) post-infection [12–20]. Two forms of mature virus particles are produced in infected cells: budded virus is released from the cell from about 8 hours post-infection [21, 22] and is responsible for cell-to-cell spread within the infected caterpillar. Occluded virus is produced later in infection and is the form of the virus preserved in polyhedra, and is responsible for infection of the next host.

With a few exceptions early in infection, there is a global down-regulation of cellular transcription in baculovirus-infected cells [23–25]. The molecular basis for how this is mediated is unclear, but given the number of genes affected (mRNA corresponding to >10,000 ESTs is transcriptionally downregulated within 12 hours post-infection [25]), this is unlikely to be a targeted programme of downregulation. The consequence of transcriptional shutdown is that cellular protein levels deteriorate in baculovirus infections between 10–12 hours postinfection and cell lysis usually occurs sometime after 76 hours postinfection. AcMNPV overcomes this transcriptional downregulation by expressing its own DNA-dependent RNA polymerase complex, which is responsible for the transcription of late and very late genes [26–33]. Coupled to a high gene copy number as a result of the replication of the virus genome, this polymerase potentiates high level synthesis of recombinant protein. The polyhedrin protein accumulates to high levels in infected cells and polyhedra can represent up to 30% of the dry weight of infected caterpillars [34]. A lot of the initial and continuing interest in baculoviruses is related to their potential as environmentally safe, targeted, biopesticides for the control of insect pests [34–37]. However, AcMNPV and BmNPV are also widely used in academic and industrial research and biopharmaceutical production.

### 1.2. Production of Recombinant Proteins in the Baculovirus System

Early experiments in protein expression using the baculovirus genome as a vector were based on the replacement of the coding sequence for the polyhedrin protein with another gene. This approach has two advantages; firstly, it capitalises on the very high expression level usually observed for polyhedrin. Secondly, as polyhedra are readily observed under a light microscope in cells that express native polyhedrin, recombinant viruses can be selected based on a polyhedra negative phenotype, allowing for selection of pure recombinant virus stocks. As the virus genome was too large for routine direct manipulation at that time, early protein expression studies took advantage of the fact that homologous recombination in baculovirus-infected insect cells is relatively efficient. Replication of the baculovirus genome involves expression of proteins that promote homologous recombination [38, 39]. Transfer vectors were constructed in which an expression cassette was flanked with regions homologous to sequences in the baculovirus genome. Cotransfection of the transfer vector and virus DNA into insect cells allowed selection of recombinant virus. The first recombinant protein successfully expressed to high levels using this approach was human beta interferon [40]. Subsequent modifications to this system included the insertion of the *LacZ *coding sequence into the polyhedrin locus of the virus genome to allow blue-white screening and linearization of the genome in an adjacent essential gene; both modifications facilitated selection of recombinant viruses [41–43]. The use of dual promoter vectors for the expression of *LacZ,* another protein from the polyhedron, and one other genetic locus (p10 locus) within the virus genome was also demonstrated [44–46].

**Figure 2:**
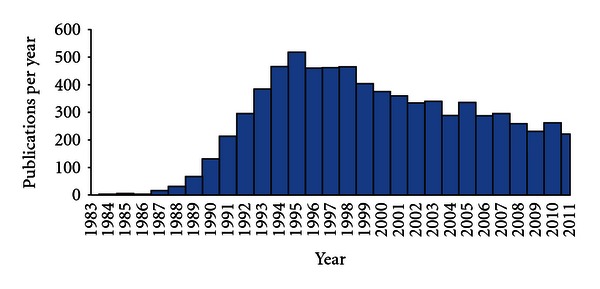
Annual publications 1983–2011 containing the search terms baculovirus or AcMNPV and recombinant protein.

Perhaps the best demonstration of the utility of recombinant protein production using the baculovirus system is by its widespread use. A search of PubMed with the terms “((baculovirus OR AcMNPV) AND recombinant protein)” returns 7281 peer-reviewed publications, between the initial description of human interferon expression in 1983, and December 2011 (Figure 2). There are now several other eukaryotic systems that are used for the expression of recombinant proteins but the baculovirus system remains a popular choice with 233 and 261 publications with the same search terms in 2009 and 2010, respectively. Recombinant proteins expressed range from small peptides to multiprotein complexes assembled from multiple subunits [47–50]. As expression is in eukaryotic cells, recombinant proteins have authentic posttranslational processing with respect to phosphorylation, palmitoylation, isoprenylation, myristoylation, and folding [51–54]. Glycoproteins expressed in the system are also usually modified by the addition of carbohydrates by the N-glycosylation pathway, but the insect cells used generally lack the capability to synthesize the terminal highly branched galactose and sialic acid carbohydrates found on proteins expressed in mammalian cells [55]. These problems can be overcome to a certain extent by using modified cells engineered to express enzymes in the human glycosylation pathway [56–58].

## 2. Early Use of Bacterial Artificial Chromosomes in the Baculovirus System

While the selection of viruses produced by homologous recombination in insect cells was a highly effective way to recover recombinant viruses, it required a reasonable level of training in virology in order to be able to complete routinely. The development of baculovirus genomes in which a bacterial artificial chromosome (BAC) sequence and transposon target site were inserted at the polyhedrin locus in the virus genome allowed the propagation of the virus genome in *E. coli* as a bacmid [59]. This system allowed the transposition of a short sequence of DNA containing the promoter for the polyhedrin gene, a coding sequence, and a polyadenylation signal sequence into the BAC DNA located at the polyhedrin locus of the AcMNPV genome. The system was designed such that successful transposition interrupted the coding sequence of the LacZ*α*  fragment, allowing selection of recombinants in *E. coli* by standard blue-white screening. This removed the need for plaque selection of recombinant viruses, which made the system more accessible to non-virology laboratories. The bacmid system was successfully marketed by Invitrogen as “Bac-to-Bac” and has been adopted by many laboratories as a method for the efficient production of recombinant baculovirus. Notable variations on this core technology include the introduction of an antibiotic resistance gene expressed in *E. coli* with the transposed element, and the inclusion of the *sacB* gene on the transfer vector, to select for recombinant bacmid and counter-select the transfer vector which has not recombined, respectively [60]. These variations allow high-throughput selection of recombinants produced using the core approach.

Since the initial description of application of the bacmid technology to AcMNPV, the approach has since been applied to other related baculoviruses [61–64]. In most cases, the role of the BAC modified virus genome has been to provide a stable reference genome for sequencing studies. But in the case of BmNPV, a similar protein expression system to the more widely used AcMNPV system has also been developed. 

## 3. Recent Developments in Baculovirus Research Enabled by BACs

### 3.1. Improved Understanding of Baculovirus Biology

In addition to facilitating insertion of foreign coding sequences into the baculovirus genome, maintenance of the virus genome as a bacmid in *E. coli* has also enabled the use of advanced reverse engineering approaches. In particular, the use of the recET/lambda-red-based recombineering approach [65] has been enthusiastically adopted by laboratories frustrated with the problem of producing null mutants in virus genes using conventional approaches. The core problem is that disruption of scientifically interesting genes often results in the production of a virus that has low or no viability. With conventional techniques, the generation of mutations in these genes either relied on conditional mutants [66, 67] or recombination in insect cells where mutations were complemented with transgenes in the virus or cellular genome [8, 68, 69]. Although effective, these approaches were time consuming and, in some cases, failed to recover the required mutation [70].

The great advantage of the BAC platform as a basis of mutagenesis is that recovery of the mutant genome does not rely on the ability of the virus to replicate in insect cells, only on the viability of the BAC sequence. Therefore, there is a similar potential for mutagenesis by homologous recombination as in insect cells, but it is possible to separate out failure of the resulting virus to replicate from failure of the recombination reaction. Bideshi and Federici first reported application of recET-based recombination to generate deletion mutants in the AcMNPV bacmid [71] using the *E. coli * strain BJ5183 [72]. This mutation involved the disruption of the AcMNPV helicase gene; subsequent studies have used similar approaches for the disruption of a range of genes involved in a number of other virus functions (Table 1). The approach has been particularly useful to characterise mutations involved in virus assembly and cell-to-cell spread, where release of the infectious virus can be separated from genome replication. 

To date, ~25% (40 out of an estimated 156) AcMNPV genes have been disrupted using the ET recombination method, demonstrating its utility for the routine modification of viral genomes maintained as bacmids. Similar approaches have been used with BmNPV [73, 74] and with other dsDNA viruses that can be maintained as bacmids in *E. coli*, including herpesviruses [75–81]. Therefore, the BAC recET system is becoming a valuable approach that has been used to aid in the understanding the mechanism of infection, assembly, replication, and host interaction of a broad range of different viruses. 

### 3.2. Improved Quality of Biopharmaceuticals

One of the problems with using baculovirus-based systems for the production of biopharmaceuticals has been the consequence that a live virus is used in the protein production process and must be purified away from any recombinant protein produced. Although baculoviruses are naturally present at high quantities in the environment and AcMNPV is easily inactivated with nonionic detergents and binary ethylenimine [82] and does not replicate in vertebrate cells [83, 84], removal of baculovirus and its DNA from protein products is generally required prior to licensing. One recent approach used an insect cell line expressing AcMNPV VP80 from a transgene integrated into the cellular chromosomal DNA and a bacmid-derived AcMNPV *vp80* null mutant [22]. As VP80 is essential for the maturation of the virus nucleocapsid in the nucleus [85], the null mutant was only able to produce viable progeny viruses in the complementing cell line. This cell line was used for amplification of the viruses and a nontransgenic cell line used for protein expression to recover recombinant protein free of viable baculovirus.

### 3.3. Recombineering for High-Throughput Production of Proteins

In addition to improving understanding of the virus biology, the bacmid platform, coupled to recET mutagenesis, has also been applied to improving the baculovirus system for protein production. Although many recombinant baculoviruses capable of expressing recombinant protein have been generated with the BAC-Tn7 transposase-based system [86], there are indications that genes inserted into the polyhedrin locus of the virus genome using this system are unstable, with recombinant protein expression declining with passage number [61, 87]. Although this can be addressed by genetic linkage of transgenes with sequences that promote retention of the foreign gene sequences, [88, 89] some laboratories maintain that insertion of genes directly into the AcMNPV genome in insect cells results in more stable protein expression. One of the problems with the latter approach has always been that recombination in insect cells required both a viable virus genome and a suitable transfer vector. Therefore, the viable virus genome inevitably led to a proportion of parental virus genomes that did not express the foreign gene and the approach was not suitable for high-throughput protein production. 

The problem of selection of recombinant viruses was partially solved by Kitts et al. [42, 90], who developed techniques allowing selection of recombinant baculovirus using linearised virus DNA and *lacZ* expression. However, although it was a significant improvement on previous approaches, there was still a residual background of replicating viruses. The problem was finally solved by Zhao et al. [91], who used the bacmid-based recET recombineering system to make a deletion in an essential gene (*orf1629*) for virus replication in insect cells that is located next to the polyhedrin locus. This mutation was only possible because the defective viral genome could be maintained in *E. coli* as a bacmid. As the virus genome could only replicate in insect cells once recombination with the transfer vector had occurred, the problem of background virus replication was eliminated. In practice, there is probably a low level of replication of the defective genome through complementation unless virus genomes are purified by plaque-cloning methods. However, this is not significant enough to interfere with most high-throughput applications. Recent developments to this approach have combined the knockout deletion in *orf1629* with deletions of *chiA*, *v-cath*, and *p10* genes, with the aim of improving expression levels and trafficking of recombinant proteins [92, 93]. These modified defective genomes are commercially available as flashBac (Oxford Expression Technologies, UK) and BacMagic (Merck).

## 4. Engineering Stable Expression of Protein Complexes

Although many protein expression experiments focus on the production of one specific recombinant protein, there is an increasing recognition that many, if not most, biological processes are completed by complexes formed of several interacting proteins. In response, there is an increasing focus on the expression and purification of these complexes using recombinant systems. The AcMNPV based-baculovirus expression system has proved to be particularly useful for the expression of functional complexes [49, 122–124]. As a eukaryotic system, baculovirus-based expression has all the appropriate chaperones and other posttranslational modification activities that facilitate the assembly of large and complex protein assemblies. 

The most straightforward approach to the production of protein complexes using the baculovirus system is the co-infection of the same batch of cells with two or more recombinant baculoviruses, each expressing a different foreign protein. The principle is that cells coinfected with the two viruses will express all the foreign proteins, allowing assembly of the complex. This approach has been used successfully for the production of many complexes and is often used to assess the potential for proteins to interact to form complexes [125–128]. However, as a method to reproducibly synthesize optimal amounts of recombinant protein, it is reliant on careful titering of infectious virus and cell numbers as the relative proportion of individual viruses present in each cell is determined by the Poisson distribution. This, in effect, means that a batch of cells infected with multiple viruses actually consists of multiple subpopulations in which the ratio of expression of different subunits in the complex varies. Furthermore, there is evidence that the Poisson distribution alone may not be sufficient to explain all variation in the level of subunit expression, and that infection with large numbers of different viruses per cell may inhibit protein expression [129]. An approach that circumvents these problems is to express multiple proteins from the same virus backbone so that all infected cells produce the recombinant proteins at the same relative ratio. Transfer vectors allowing the expression of multiple proteins from the same baculovirus genome were first constructed some time ago [130–133]. However, due to the need to insert multiple genes into the same vector, and the fact that the upper size of the vector restricts the number of genes that can be inserted, cloning strategies for these vectors, especially for large complexes, are not straightforward.

The first attempt to address this problem, based on the manipulation of bacmid DNA was by Berger et al. [134]. In these experiments, a *LoxP* recombination target site was inserted at the position of the *chiA *and *v-cath* genes that are adjacent in the baculovirus genome in the bacmid, using the recET recombineering approach. As the bacmid already contained the Tn7 transposase target site, the *LoxP* site allowed insertion of foreign genes at a second locus by Cre-mediated recombination. The resulting system is referred to as “Multibac” in the literature. The advantage of this system is that it is possible to “mix and match” the sequences inserted at the polyhedrin and *chiA-v-cath *loci [135, 136]. By combining with transfer vectors that are modified to allow repeated insertions of dual expression cassettes by conventional ligation or ligation-independent cloning approaches, expression of multiple protein subunits from the same baculovirus is possible [134, 135]. This partially solves the problem of large transfer vectors as genes are split between two loci, effectively doubling the limit imposed by maximum transfer vector size. Furthermore, as the system was developed specifically to allow automation of protein production by robotics, many different combinations of subunits can be rapidly tested for any one complex, which is particularly useful for structural studies [135]. Although in principle this system can be used for the expression of protein complexes with a large number of different subunits, early experiments suggested that there was some genetic instability, possibly due to repeated DNA elements [136]. This was not supported in subsequent experiments which suggested that 5 different recombinant proteins were still expressed after 4 low multiplicity of infection *in vitro *passages in 90% of cells [135]. However, studies on transposon insertions with a single insert predict that effects of gene deletion in baculovirus experiments may not be apparent before 5–10 passages anyway [87]. Overall, there is no clear evidence that inserts in the modified system are any less stable than the original Tn7 transposase-based bacmid system [59]. 

**Figure 3:**
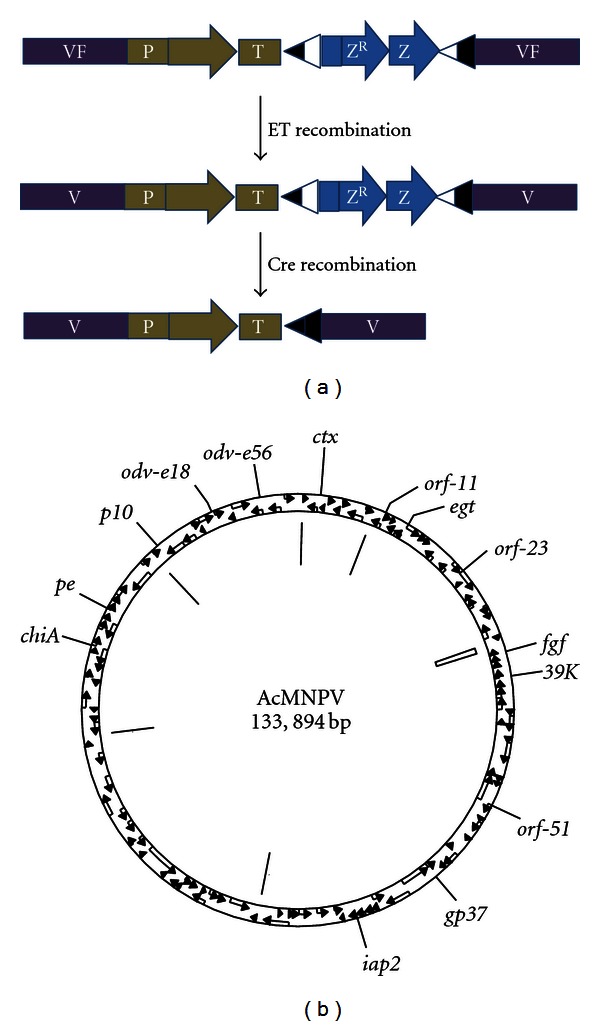
Iterative modification of AcMNPV to insert multiple single-locus expression cassettes. (a) Strategy used for repeated modification of the same bacmid to express multiple different recombinant proteins. ET recombination is targeted by viral flanking sequences (VF) to homologous sequences in the bacmid. Selection in *E. coli* is achieved using a bipartite marker consisting of a Zeocin resistance (Z^R^) and LacZ*α* (Z), flanked by partially defective loxP sites. Following Cre-mediated recombination, this marker is removed, allowing a subsequent modification of the same DNA. (b) Loci within the AcMNPV successfully modified using the iterative modification strategy.

An alternative system for the production of multiprotein complexes from baculovirus genomes engineered to co-express protein subunits avoids both the transfer vector size problem and the potential for genetic instability in insect cells [47]. Instead of using site-specific transposase or recombinase activities to integrate genes into the baculovirus genome, the approach takes advantage of improved selection of recombinants to use the recET recombineering approach to directly insert cassettes for foreign gene expression at different genetic loci in the baculovirus genome (Figure 3). 

In order for the BAC-based recET approach to be viable for the routine insertion of genes for protein expression, it was necessary to significantly improve the ease with which recombinants could be selected. To achieve this, a bipartite selection system that allowed at least a 21-fold increase in the efficiency of selection of recombinants was developed [47]. By coupling this selection to self inactivating loxP sites, it was possible to achieve iterative modifications at multiple sites in the same genome using the same selection. Using this system coexpression of protein complexes containing two, four, and different recombinant proteins was demonstrated.

## 5. Conclusions and Future Perspectives

The use of bacterial artificial chromosomes continues to have a major impact on the development of studies for baculovirus and other large DNA viruses. Coupled to the recET recombineering approaches [65, 137, 138], the BAC platform is a powerful tool for understanding both the basic biology and for developing the industrial applications of baculovirus systems. The combination of null mutants with complementation has provided interesting insights into the role of viral proteins in virus replication, host interaction, and assembly. Current studies have been relatively crude and have resulted in deletions in the virus genome. As genes are arranged in both directions on the circular dsDNA AcMNPV genome, the impact of these deletions on flanking genes is a potential problem. Future studies may very well involve more seamless modification of the virus genome.

In terms of the applied use of baculovirus systems, BACs have facilitated protein expression for a number of years. The new recombineering approaches have also opened up new ways to produce virion-free biopharmaceuticals [22], and for the efficient production of multiprotein complexes [47, 134]. However, these are only beginning to scratch the surface of what is now possible with the baculovirus system. The demonstration that the routine integration of transgenes at any locus in the AcMNPV genome is now possible [47] opens the possibility of modifying background genomes to facilitate the expression environment for specific recombinant proteins. 

## Figures and Tables

**Table 1:** Summary of null mutants in AcMNPV generated in BACmid DNA by ET recombination.

Virus function	Gene deleted	References
DNA replication	*helicase (p143), lef3, lef5, lef6, lef11, alk-exo, dna-pol*	[70, 71, 94–100]
Transcription	*ie-0 *and *ie-1 *	[101]
Cell entry	*ac23, gp64*	[47, 102, 103]
Infectivity in insects	*ac96, p74*	[104, 105]
Nucleocapsid assembly	*p6.9, ac53, vlf-1, 38K, ac101, ac142, ac144, ac109*	[106–112]
Nucleocapsid release from the nucleus	*exon0, ac66, vp80*	[22, 85, 113–115]
Formation of enveloped virus	*ac142, p48, ac76, odv-e18, odv-e56*	[47, 116–118]
Interaction with host	*egt, chiA, v-cath, IAP2*	[47]
Unknown, essential genes	*ac92, ac-fgf, ac11, ac-pe*	[47, 119]
Unknown, non-essential genes	*me53, ac18, ac-ctx*	[47, 120, 121]
